# Overlapping profiles of Aβ peptides in the Alzheimer's disease and pathological aging brains

**DOI:** 10.1186/alzrt121

**Published:** 2012-05-23

**Authors:** Brenda D Moore, Paramita Chakrabarty, Yona Levites, Tom L Kukar, Ann-Marie Baine, Tina Moroni, Thomas B Ladd, Pritam Das, Dennis W Dickson, Todd E Golde

**Affiliations:** 1 Center for Translational Research in Neurodegenerative Disease, Department of Neuroscience, McKnight Brain Institute, College of Medicine, University of Florida, 1275 Center Drive, Gainesville, FL, 32610, USA; 2 Department of Pharmacology, Emory University School of Medicine, 1510 Clifton Road, Atlanta, GA, 30322, USA; 3 Department Of Neuroscience, Mayo Clinic College of Medicine, 4500 San Pablo Road, Jacksonville, FL, 32224, USA

## Abstract

**Introduction:**

A hallmark of Alzheimer's disease (AD) is the presence of senile plaques composed of aggregated amyloid β (Aβ) peptides. Pathological aging (PA) is a postmortem classification that has been used to describe brains with plaque pathology similar in extent to AD, minimal cortical tau pathology, and no accompanying history of cognitive decline in the brain donor prior to death. PA may represent either a prodromal phase of AD, a benign form of Aβ accumulation, or inherent individual resistance to the toxic effects of Aβ accumulation. To attempt to distinguish between these possibilities we have systematically characterized Aβ peptides in a postmortem series of PA, AD and non-demented control (NDC) brains.

**Methods:**

Aβ was sequentially extracted with tris buffered saline (TBS), radioimmunoprecipitation buffer (RIPA), 2% sodium dodecyl sulfate (SDS) and 70% formic acid (FA) from the pre-frontal cortex of 16 AD, eight PA, and six NDC patients. These extracts were analyzed by 1) a panel of Aβ sandwich ELISAs, 2) immunoprecipitation followed by mass spectrometry (IP/MS) and 3) western blotting. These studies enabled us to asses Aβ levels and solubility, peptide profiles and oligomeric assemblies.

**Results:**

In almost all extracts (TBS, RIPA, 2% SDS and 70% FA) the average levels of Aβ1-40, Aβ1-42, Aβ total, and Aβx-42 were greatest in AD. On average, levels were slightly lower in PA, and there was extensive overlap between Aβ levels in individual PA and AD cases. The profiles of Aβ peptides detected using IP/MS techniques also showed extensive similarity between the PA and AD brain extracts. In select AD brain extracts, we detected more amino-terminally truncated Aβ peptides compared to PA patients, but these peptides represented a minor portion of the Aβ observed. No consistent differences in the Aβ assemblies were observed by western blotting in the PA and AD groups.

**Conclusions:**

We found extensive overlap with only subtle quantitative differences between Aβ levels, peptide profiles, solubility, and SDS-stable oligomeric assemblies in the PA and AD brains. These cross-sectional data indicate that Aβ accumulation in PA and AD is remarkably similar. Such data would be consistent with PA representing a prodromal stage of AD or a resistance to the toxic effects of Aβ.

## Introduction

Alzheimer's disease (AD) is characterized by large numbers of extracellular amyloid plaques with dense amyloid cores that are associated with dystrophic neurites and neuroinflammatory changes as well as intraneuronal neurofibrillary tangles. Pathological aging (PA) patients also have abundant and widespread amyloid plaques; however, these plaques have typically been described as diffuse in nature. In PA there are fewer cored plaques and there is little or no inflammatory reaction, neuritic pathology or neurofibrillary tangles in the cortex. These patients are reported to be cognitively normal prior to death [1-3]. Based on our current understanding of the progression of AD, PA may represent a prodromal phase of AD (for example, preclinical stage 1 AD, plaque only), a benign form of Aβ accumulation, or inherent individual resistance to the toxic effects of Aβ accumulation [3,4].

Aβ is the principle component of amyloid deposits in the AD brain. It is a secreted peptide produced through sequential cleavage of the Amyloid-β Protein Precursor (APP) by β- and γ-secretases [5-7]. Aβ peptides have a heterogeneous carboxyl-terminus with the majority (approximately 40% to 70%) composed of 40 amino acids Aβ1-40, while a minor product (approximately 5% to 20%) contains a two amino acid extension Aβ1-42. Additional minor Aβ peptides are also normally produced (for example, 1-34, 1-37, 1-38 and 1-39), although few reports have quantified the levels of these peptides in the brain [8]. Aβ1-42 is more amyloidogenic and has been implicated as the pathogenic form of Aβ [9]. A recent study also suggested that Aβ1-43 could play a critical role in Aβ accumulation [10]. Furthermore, a variety of truncated and modified Aβ peptides have been described (for example, 1-28, 1-29, 1-45, 2-46, 3-44, 3-47, 2-42, 4-42, 5-42, 6-42, 7-45, 8-42, 1-42Met35ox, pE3-42, pE11-42) [11-18]. Of these truncated and modified forms the pyroglutamate modified forms, AβpE3-42 and AβpE11-42, have been highly investigated, as key species possibly involved in initial nucleation or seeding events [19-22].

Once liberated from APP, Aβ can self-associate to form various aggregates. These aggregates include soluble oligomers, protofibrils, and amyloid fibrils [23,24]. Although there is currently debate within the field regarding which form or forms of Aβ aggregates are the most pathogenic, there is general consensus that the aggregated forms of Aβ are harmful and that Aβ1-42 or possibly Aβ1-43 is required for aggregation in the absence of internal mutations within Aβ [25-30].

Because many different forms of Aβ exist and accumulate in various higher order assemblies, it is possible that the relatively poor correlation between cognitive deficits and plaque load is attributable to either qualitative or quantitative differences between a particular species or assembly of Aβ. This poor correlation could also reflect an inherent difference in vulnerability to 'toxic' effects of different forms of Aβ aggregates. Additionally, given the growing acceptance of the concept of preclinical AD [4,31,32], where the initial stage is defined by the presence of Aβ deposits in the absence of other pathologies and no evidence for cognitive impairment, the poor correlation could be attributable to differences in time from initial deposition to frank neurodegeneration and clinical deterioration.

As PA represents the most clear cut example of the dissociation between Aβ accumulation and cognitive impairment, investigation of the type and species of Aβ peptides in PA cohorts could provide novel insights into the poor correlation between Aβ and cognition. Previous studies investigating differences in Aβ1-40 and Aβ1-42 species extracted from PA and AD brains demonstrated that Aβ1-40 levels were as much as approximately 20-fold higher in AD brains compared to PA brains whereas Aβ1-42 levels were only about 2-fold higher [33]. A more recent and extensive study using both ELISAs and western blotting to analyze Aβ levels and oligomeric assemblies failed to detect major differences in PA and AD [34]. Other more anecdotal studies comparing oligomeric assemblies in a single PA brain versus AD brains failed to detect significantly elevated levels of Aβ dimer in the PA brain extracts compared to AD brain extracts [35].

Although these studies suggest that there may be both quantitative and qualitative differences in Aβ peptides in PA brains as opposed to AD brains, we felt that a more extensive investigation with larger cohorts was warranted. Here we report on our analysis of Aβ peptides sequentially extracted from the pre-frontal cortices of 16 AD patients, eight PA patients, and six non-demented controls (NDC) using a battery of biochemical tests. Our analysis shows that AD and PA brains are clearly distinct from controls, but there is extensive overlap between PA and AD with respect to extractable Aβ levels as measured by ELISA. Using immunoprecipitation mass spectrometry (IP/MS) to profile individual Aβ species in the PA and AD brain extracts we find that there is also extensive overlap in the profiles of accumulated Aβ. However, individual AD brains showed more extensive heterogeneity with an increase of diversity of Aβ species, particularly amino-terminally truncated Aβ species. Assessment of SDS-stable oligomers by western blotting also showed no consistent differences between PA and NDC.

## Materials and methods

### Selection of PA cases

Frozen pre-frontal cortex (AD = 16, PA = 8, NDC = 7) was obtained from the Mayo Clinic Brain Bank with informed consent, in accordance with the Mayo Clinic institutional review board, using previously described acquisition and diagnostic analyses [2,36,37]. We analyzed 16 brains from AD patients (age range = 66 to 99; average age = 82), eight pathologic aging brains from subjects (age range = 66 to 90; average age = 80) without clinical evidence of dementia and seven brains with rare or no AD lesions from elderly individuals without clinical evidence of a neurological illness (age range = 66 to 87; average age = 76). Table 1 summarizes the cases studied here.

**Table 1:** Summary of demographics of human subjects studied.

	Group		
	**AD (*n *= 16)**	**PA (*n *= 8)**	**NDC (*n *= 7)**

Age	82 (66 to 99)	80 (66 to 90)	76 (66 to 87)
Gender			
M	8 (50%)	6 (75%)	5 (71%)
F	8 (50%)	2 (25%)	2 (29%)
Braak			
2.0	0 (0%)	4 (50%)	4 (57%)
2.5	0 (0%)	1 (13%)	1 (14%)
3.0	0 (0%)	3 (38%)	2 (29%)
3.5	0 (0%)	0 (0%)	0 (0%)
4.0	2 (13%)	0 (0%)	0 (0%)
4.5	2 (13%)	0 (0%)	0 (0%)
5.0	7 (44%)	0 (0%)	0 (0%)
5.5	2 (13%)	0 (0%)	0 (0%)
6.0	3 (19%)	0 (0%)	0 (0%)
CAA			
0	6 (38%)	5 (63%)	5 (71%)
0 - 1+	1 (6%)	0 (0%)	1 (14%)
1+	6 (38%)	0 (0%)	1 (14%)
1+ - 2+	1 (6%)	0 (0%)	0 (0%)
2+	1 (6%)	1 (13%)	0 (0%)
2+ - 3+	0 (0%)	1 (13%)	0 (0%)
3+	0 (0%)	1 (13%)	0 (0%)
4+	1 (6%)	0 (0%)	0 (0%)

Median ages (age range), gender distribution, Braak score and CAA score are denoted. CAA scoring was done by post-mortem neuropathologic analysis by DW. AD, Alzheimers disease; CAA, cerebral amyloid angiography; F, female; M, male; n, number; NDC, non-demented controls; PA, Pathological aging.

### Brain extraction

Frozen pre-frontal cortex tissue was cryo-pulverized in liquid nitrogen. Briefly, for ELISA and IP/MS, the cryo-pulverized tissue was sequentially extracted with Tris-buffered saline (TBS), radioimmunoprecipitation buffer (RIPA), 2% sodium dodecyl sulfate (SDS) and 70% formic acid (FA) containing protease inhibitor cocktail (Roche, Indianapolis, IN, USA) as described before at a concentration of 300 mg/mL [38]. For immunoblotting, samples were either serially extracted in TBS and 2% SDS or directly extracted with RIPA at a concentration of 500 mg/mL.

### ELISA

TBS, RIPA, 2% SDS and neutralized 70% FA extracted samples were diluted appropriately and used for sandwich ELISAs as described previously [39]. Aβ1-40 was captured with monoclonal antibody (mAb) Ab9 (human Aβ1-16 specific; T.E. Golde) and detected by horseradish peroxidase (HRP)-conjugated mAb 13.1.1 (human Aβ35-40 specific; T.E. Golde); Aβ1-42 was captured with mAb 2.1.3 (human Aβ35-42 specific; T.E. Golde) and detected by HRP-conjugated mAb Ab9; Total Aβ was captured with mAb Ab9 and detected by HRP-conjugated mAb 4G8 (Covance, Princeton, NJ, USA); Aβx-42 was captured with mAb 2.1.3 and detected by HRP-conjugated mAb 4G8.

### Immunoprecipitation followed by mass spectrometry (IP/MS)

Magnetic sheep-anti-mouse IgG beads (Invitrogen, Grand Island, NY, USA) were incubated with 4.5 μg antibody (either Ab9 or 4G8 (Covance)) for 30 minutes at room temperature with constant shaking. The beads were then washed and incubated with each extract, which were diluted appropriately. All sample incubations were in the presence of 0.1% TritonX-100 (Tx-100) and either 10 pmol Aβ1-28 (TBS and RIPA extracts) or 100 pmol Aβ1-28 (2% SDS and 70% FA extracts) as an internal calibration standard. The samples were successively exposed to Ab9 and 4G8 coated beads for 30 minutes each with rotation. Bound beads were washed sequentially with 0.1% and 0.05% Tx-100 followed by water. Samples were eluted using a mix of 75% acetonitrile, 24.9% water and 0.1% FA. Samples were mixed in equal volume with sinapinic acid (25 mg/mL) in 50% acetonitrile, 49.5% water and 0.5% trifluoroacetic acid, and 1 μL was spotted onto a ProteinChip Gold Array (A-H format) (Bio-Rad Hercules, CA, USA) and analyzed with a Bio-Rad ProteinChip System Series 4000 (Enterprise Edition) mass spectrometer.

### Western blotting

TBS, RIPA and 2% SDS brain lysates, heated at 50°C for three minutes in the presence of denaturing sample buffer, were separated on 4% to 12% Bis-Tris gel (Bio-Rad) in 1X 2-(N-morpholino)ethanesulfonic acid (MES) running buffer (Bio-Rad). Initially, we performed a comparative analysis of different immunoblotting techniques with different combinations of membranes (0.2 μm nitrocellulose and 0.2 μm polyvinylidene fluoride (PVDF)) and antigen retrieval techniques (boiling, glutaraldehyde and guanidine) in combination with three different primary antibodies (4G8, 82E1 (IBL, Hamburg, Germany) and Ab5 (human Aβ1-16 specific; T.E. Golde). After extensive analysis samples immunoblotted with 82E1 on boiled nitrocellulose membranes yielded the best results in terms of sensitivity and resolution. Transferred membranes were blocked in Starting Block (Thermo Scientific, Waltham, MA) and incubated overnight with primary antibody (82E1) and detected with donkey anti-mouse antibody conjugated to HRP (Jackson ImmunoResearch, West Grove, PA). Chemilumiscence signal (West Femto Chemiluminescent Substrate (Thermo Scientific)) was visualized with a FujiFilm system.

### Statistical analysis

Results, unless otherwise mentioned, were analyzed with Prism 5 (GraphPad) by one way analysis of variance (ANOVA) with tukey *post hoc *test and presented as data ± standard error of the mean (s.e.m.). Statistical significance is denoted by an asterisk.

## Results

### Immunohistochemical characterization of Aβ plaques and phospho-tau in AD, PA and NDC

**Figure 1:**
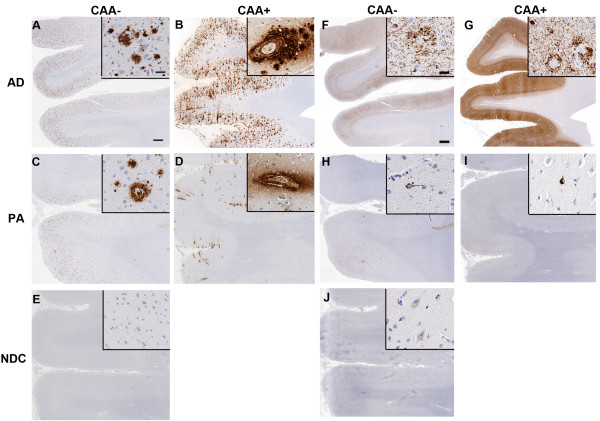
**Immunohistochemical characterization of amyloid and tau pathology in Alzheimer's disease (AD), pathological aging (PA) and normal non-demented controls (NDC)**. **A-E**. Representative paraffin embedded formalin fixed hippocampi from human AD (A, B), PA (C, D) and control (NDC; E) subjects were stained with 33.1.1 antibody (pan Aβ1-16). Insets show higher magnification with special emphasis on cored Aβ plaques associated with blood vessels in subjects with a post-mortem clinical pathological diagnosis of cerebral amyloid angiopathy (CAA+). *Scale Bar*, 600 μm (A-E), *insets *60 μm (A-E). **F-J**. Representative paraffin embedded formalin fixed hippocampi from human AD (F, G), PA (H, I) and NDC (J) subjects were stained with anti-phosphorylated tau (CP13). Insets highlight phorphorylated tau containing tangles and neuritic structures. *Scale Bar*, 600 μm (A-E), *insets *60 μm (A-E). Aβ, amyloid β.

**Figure 2:**
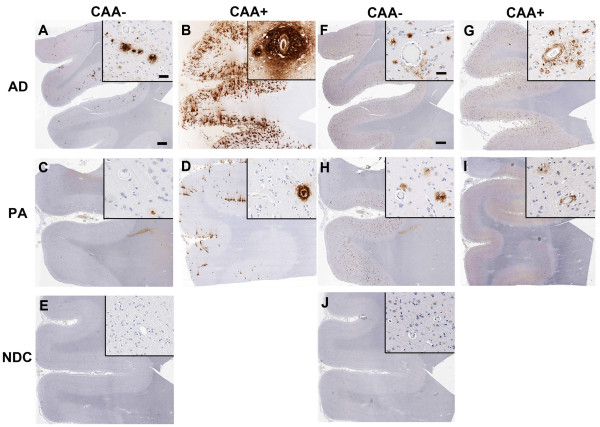
**Immunohistochemical characterization of Aβ1-40 and Aβ1-42 in Alzheimer's disease (AD), pathological aging (PA) and normal non-demented controls (NDC)**. **A-E**. Representative paraffin embedded formalin fixed hippocampi from human AD (A, B), PA (C, D) and control (NDC; E) subjects were stained with 13.1.1 antibody (Aβ35-40). Insets show higher magnification with special emphasis on cored Aβ plaques associated with blood vessels in subjects with a post-mortem clinical pathological diagnosis of cerebral amyloid angiopathy (CAA+). *Scale Bar*, 600 μm (A-E), *insets *60 μm (A-E). **F-J**. Representative paraffin embedded formalin fixed hippocampi from human AD (A, B), PA (C, D) and control (NDC; E) subjects were stained with 2.1.3 antibody (Aβ35-42). Insets show higher magnification with special emphasis on cored Aβ plaques associated with blood vessels in subjects with a post-mortem clinical pathological diagnosis of cerebral amyloid angiopathy (CAA+). *Scale Bar*, 600 μm (A-E), *insets *60 μm (A-E). Aβ, amyloid β.

The sample population was chosen based on long standing AD and PA classification systems. Brains selected as PA had no evidence of cognitive decline in the clinic, but had extensive cortical Aβ plaques (Table 1). To obtain more extensive characterization of Aβ and tau pathology in the PA cohort, we immunostained frontal cortical tissue for total Aβ, phospho-tau, Aβ1-40 (13.1.1) and Aβ1-42 (21.3.1) (Figures 1, 2). Consistent with the PA classification system, PA brains had extensive amyloid deposits and displayed only sparse CP13 immunoreactivity whereas AD patients contained widespread amyloid deposits as well as abundant phospho-tau bearing neurofibrillary tangles (Figure 1, A, B, C and 1D and 1F, G, H and 1I). PA brains showed more widespread Aβ1-42 immunoreactivity than Aβ1-40 immunoreactivity, perhaps corresponding to more abundant diffuse plaques as alluded to by independent groups (Figure 2, C, D and 2H, I). Cohorts with increased vessel-associated Aβ1-40 immunoreactivity have been sub-categorized as having cerebral amyloid angiopathy (CAA); CAA+ AD patients had more Aβ1-40 than the corresponding PA group (Figure 2, A, B, C, D, F, G, H and 2I).

### Biochemical results of Aβ levels in AD, PA and NDC

**Figure 3:**
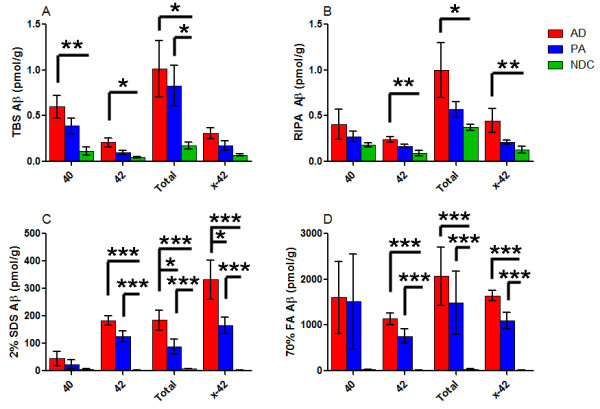
**Biochemical analysis of Aβ from human brain lysates**. Human prefrontal cortical tissue from Alzheimer's disease (AD), pathological aging (PA) and normal controls (NDC) was sequentially extracted with TBS (**A**), RIPA (**B**), 2% SDS (**C**) and 70% FA (**D**). End-specific sandwich ELISAs measuring Aβ1-40, Aβ1-42, Aβtotal and Aβx-42 are presented for each of these fractions. *N *= 16 (AD), 8 (PA) and 6 (NDC). (***P < 0.001, **P < 0.01, *P < 0.05 by ANOVA with tukey post-hoc analysis raw data analyzed (**A, B**) and log-transformed data analyzed (**C, D**)). Aβ, amyloid β; ANOVA, analysis of variance; ELISA, enzyme-linked immunosorbent assay; FA, formic acid; RIPA, radioimmunoprecipitation buffer; SDS, sodium dodecyl sulfate; TBS, Tris buffered saline.

We analyzed Aβ levels from sequentially extracted brain lysates using four different anti-Aβ antibody combinations to detect Aβ1-40, Aβ1-42, Aβtotal and NH_2_-truncated Aβx-42 species (Figure 3). In each of our Aβ ELISAs the detection limit was approximately 0.04 pmol/g. Compared to the 2% SDS and 70% FA extracted fractions, minimal Aβ was detected in the TBS and RIPA fractions. For example, in AD extracts the mean total Aβ measured was 1.0, 1.0, 184 and 2,065 pmol/g for the TBS, RIPA, 2% SDS and 70% FA extracts, respectively (Figure 3). The levels of total Aβ in PA lysates were 0.8, 0.6, 87 and 1,490 pmol/g for the TBS, RIPA 2% SDS and 70% FA extracts, respectively (Figure 3). Thus, the sequential solubility of the Aβ was similar in AD and PA. In NDC brain extracts the levels of total Aβ were 0.2, 0.4, 7 and 38 pmol/g for the TBS, RIPA, 2% SDS and 70% FA extracts, respectively (Figure 3). Thus, a major difference between AD, PA, and controls, is the dramatic increase in Aβ that requires either 2% SDS or 70% FA to solubilize, suggesting that both AD and PA cohorts had dramatically increased levels of insoluble aggregates of Aβ.

On average, AD and PA lysates exhibited much higher levels of Aβ than NDC samples, with mean Aβ levels in each PA lysate ranging from almost equivalent to approximately 50% less than the Aβ level detected in AD lysates (Figure 3). Although not reaching significance in each analysis, there were clear differences between the average levels of Aβ in AD and PA extracts as compared to NDC. There was also extensive overlap between individual Aβ levels in PA and AD as shown in Additional file 1, Figure S1.

### Profiling of Aβ peptides by immunoprecipitation/mass spectrometry (IP/MS)

**Figure 4:**
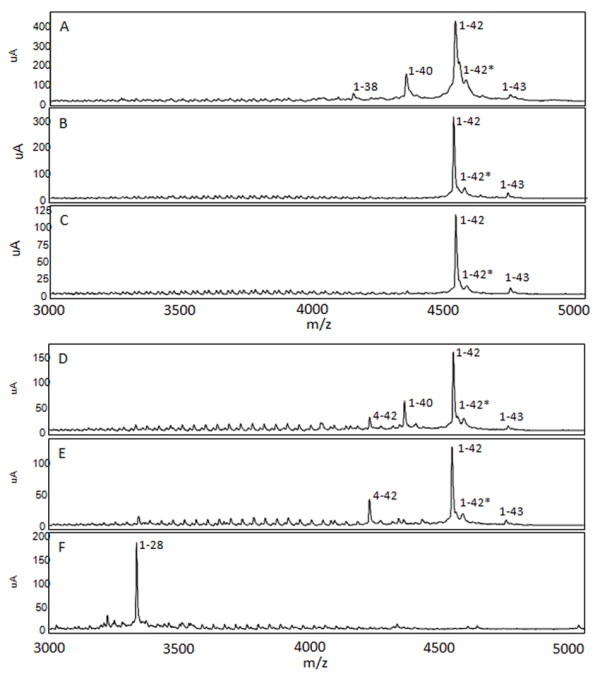
**Mass spectrometric (MS) analysis following immunoprecipiation of Aβ from 2% SDS extracted lysates of human subjects**. 2% SDS lysates of Alzheimer's disease (AD; **A, D**), pathological aging (PA; **B, E**) and control (NDC; **C, F**) subjects were subjected to immunoprecipitation with pull-down by Ab9 (A-C), sequential pull-down with Ab9 and 4 G8 (D-F). Representative MS spectra are shown (A-F). Peaks corresponding to Aβ peptides have been labeled according to m/z. Aβ1-28 was spiked in as an experimental control. The 1-42* peak denotes a possible modified Aβ1-42 species (Aβ1-42 + 16 Da, A-E). Aβ, amyloid β; SDS, sodium dodecyl sulfate.

**Figure 5:**
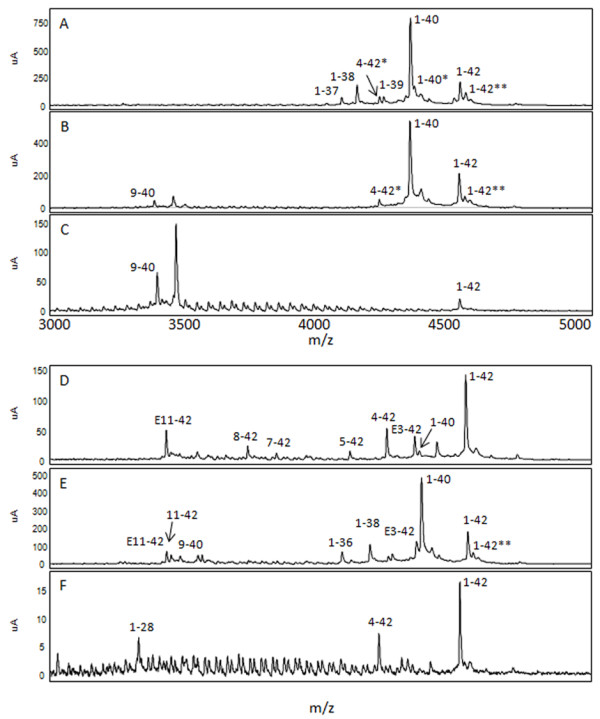
**Mass spectrometric (MS) analysis following immunoprecipiation of Aβ peptides from 70% formic acid extracted lysates of human subjects**. 70% formic acid extracted lysates of Alzheimer's disease (AD; **A, D**), pathological aging (PA; **B, E**) and control (NDC; **C, F**) cohorts were subjected to immunoprecipitation with pull-down by Ab9 (A-C), sequential pull-down with Ab9 and 4G8 (D-F). Representative MS spectra are shown. Peaks corresponding to Aβ peptides have been labeled according to m/z. Aβ1-28 was spiked in as an experimental control. Different peaks corresponding to modified Aβ are shown (4-42*, Aβ4-42 + 16 Da; 1-40*, Aβ1-40 +16 Da; 1-42**, Aβ1-42 + 22 Da). Aβ, amyloid β.

**Figure 6:**
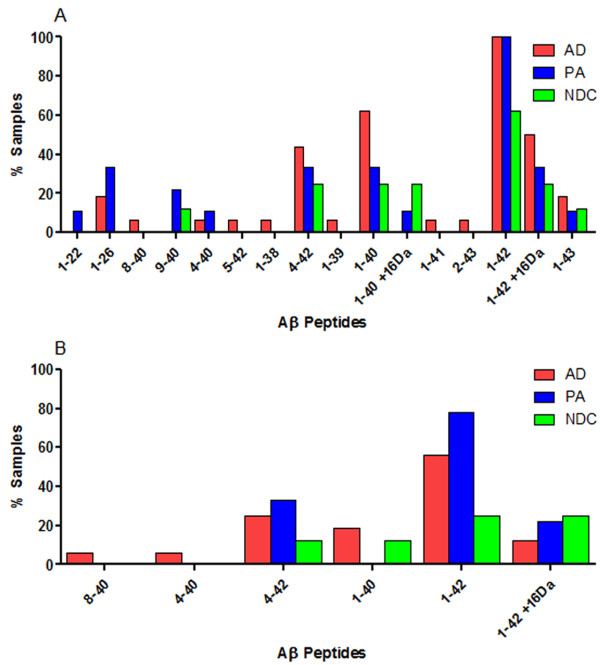
**Summary of SDS soluble Aβ peptides in test subjects**. 2% SDS extracted lysates from human prefrontal cortical tissues were subjected to immunoprecipitation with pull-down by Ab9 (**A**) or sequential pull-down with Ab9 and 4G8 (**B**), followed by MS. Data are graphically presented as a percentage of subjects in each cohort (Alzheimer's disease (AD), Pathological aging (PA) or controls (NDC)) containing Aβ peptides (x-axis). *N *= 16 (AD), 8 (PA) and 6 (NDC). Aβ, amyloid β; MS, mass spectrometry; N, number; SDS, sodium dodecyl sulfate.

**Figure 7:**
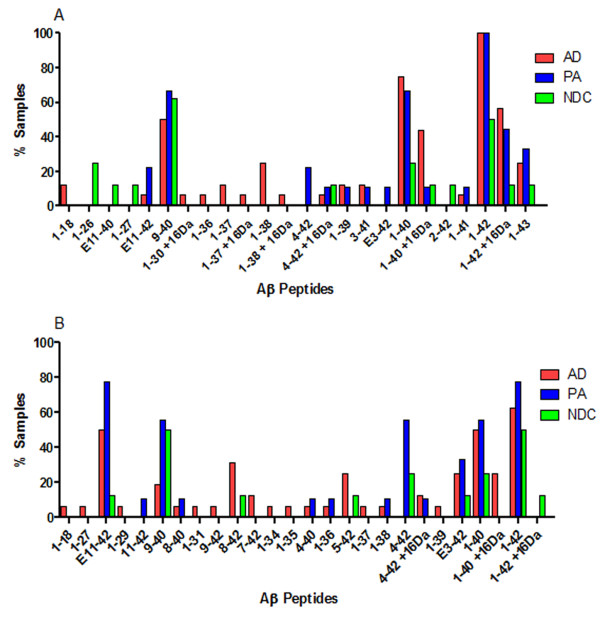
**Summary of formic acid soluble Aβ peptides in test subjects**. 70% FA extracted lysates from human prefrontal cortical tissue were subjected to immunoprecipitation with pull-down by Ab9 (**A**) or sequential pull-down with Ab9 and 4G8 (**B**), followed by MS. Data are graphically presented as a percentage of subjects in each cohort (Alzheimer's disease (AD), Pathological aging (PA) or controls (NDC)) containing Aβ peptides (x-axis). *N *= 16 (AD), 8 (PA) and 6 (NDC). Aβ, amyloid β; FA, formic acid; MS, mass spectrometry; N, number.

We next analyzed the lysates by IP/MS to identify various Aβ peptides associated with AD, PA and NDC cohorts. We used sequential immunoprecipitations using two non-overlapping anti-Aβ antibodies, Ab9 (anti-Aβ1-16) followed by 4G8 (anti-Aβ17-24), as our pilot studies suggested that predominant species (detected by Ab9) obscure less abundant peptides, and that Ab9 does not capture all NH_2_-terminally truncated Aβ peptides. Spectra from the TBS and RIPA lysates had low signal to noise ratio attributable to the low amounts (0 to 4 pmol/g) of Aβ in these samples limiting our ability to definitively identify Aβ peptides. In contrast, high quality spectra were obtained for the vast majority of the 2% SDS and 70% FA extracted lysates. Representative spectra of the 2% SDS and 70% FA extracts after immunoprecipitation with Ab9 and 4G8 are shown in Figures 4 and 5, respectively. In these spectra Aβ species were assigned to the inferred mass based on m/z. Numerous COOH- and NH_2_-terminal fragments of Aβ peptides were observed in a subset of the lysates from the AD, PA and NDC cohorts; however, few unique truncated peptides were identified in any patient group, and these 'unique' peptides were only detected in a minority of the samples in that group. Unique peptides that 1) have not been previously reported in other MS analyses of AD brains (for example, Aβ1-26, 9-40 and 4-40) or 2) that were uniquely present in PA (for example, Aβ1-22, 4-42 and 11-42), AD (for example, Aβ8-40, 1-37, 1-38 and 1-39) or NDC (for example, Aβ1-26, 1-27 and 2-42) are distinguished in Tables 2, 3, 4 and 5 by differential fonts. Though the profiles were overlapping between AD, PA and NDC samples (Figures 4, 5, 6 and 7; Tables 2, 3, 4 and 5), there were more complex mixtures of Aβ peptides observed in a subset of the AD samples versus PA and for most of the AD and PA samples relative to control. These differences typically related to minor peaks in a subset of the spectra. This did not appear to be related to absolute levels of Aβ as they were similar to levels measured by ELISAs in the starting extracts. Notably, consistent with many previous studies, Aβ1-42 was the only species consistently detected by the Ab9 IP/MS in all AD and PA brains.

**Table 2:** Mass spectrometric analysis of SDS soluble fraction immunoprecipitated with Ab9 from human brains reveal unique Aβ peaks.

Aβ Peptide	predicted MW	observed MW	AD (*n *= 16)	PA (*n *= 9)	NDC (*n *= 8)
1-22	2661.82	2663.37		*1 (11.1%)*	
1-26	3020.16	3021.17	3 (18.8%)	3 (33.3%)	
8-40	3458.98	3461.27	**1 (6.3%)**		
9-40	3371.90	3372.80		2 (22.2%)	1 (12.5%)
4-40	4014.57	4016.50	1 (6.3%)	1 (11.1%)	
5-42	4051.57	4053.50	**1 (6.3%)**		
1-38	4131.52	4133.95	**1 (6.3%)**		
4-42	4198.74	4200.44	7 (43.8%)	3 (33.3%)	2 (25%)
1-39	4230.65	4233.33	**1 (6.3%)**		
1-40	4329.78	4331.96	10 (62.5%)	3 (33.3%)	2 (25%)
1-40 +16 Da	4345.78	4345.96		1 (11.1%)	2 (25%)
1-41	4442.94	4446.86	**1 (6.3%)**		
2-43	4500.11	4503.58	**1 (6.3%)**		
1-42	4514.02	4516.48	16 (100%)	9 (100%)	5 (62.5%)
1-42 +16 Da	4530.02	4531.81	8 (50%)	3 (33.3%)	2 (25%)
1-43	4615.20	4616.60	3 (18.8%)	1 (11.1%)	1 (12.5%)

Human brain tissue was extracted with 2% SDS and sequentially immunoprecipitated with Ab9 and 4G8 antibodies to enrich different truncated or modified Aβ peptides. Mass spectrometric analysis shows unique Aβ peptides that 1) have not been previously reported in other mass spectrometric analyses of AD brains (underlined) 2) uniquely present in AD (bold font) or PA (italicized) are differentially highlighted. Predicted molecular weights and observed molecular weights have been calculated using ExPASy PeptideMass and ProteinChip Data Manager software (Bio-Rad), respectively. The number of subjects (AD, Alzheimer's disease; PA, Pathological aging; NDC, controls) displaying each uniquely identifiable Aβ peptides and the percentage of occurrence of such peptides corresponding to the total sample size is denoted. Aβ, amyloid β; MW, molecular weight; n, number; SDS, sodium dodecyl sulfate.

**Table 3:** Mass spectrometric analysis of SDS soluble fraction immunoprecipitated with 4G8 from human brains reveal unique Aβ peaks.

Aβ Peptide	predicted MW	observed MW	AD (*n *= 16)	PA (*n *= 9)	NDC (*n *= 8)
8-40	3458.98	3462.81	**1 (6.3%)**		
4-40	4014.57	4018.49	**1 (6.3%)**		
4-42	4198.74	4202.62	4 (25.0%)	3 (33.39%)	1 (12.5%)
1-40	4329.78	4333.49	3 (18.8%)		1 (12.5%)
1-42	4514.02	4516.74	9 (56.3%)	7 (77.80%)	2 (25%)
1-42 +16 Da	4530.02	4534.90	2 (12.5%)	2 (22.2%)	2 (25%)

Human brain tissue was extracted with 2% SDS and sequentially immunoprecipitated with Ab9 and 4G8 antibodies to enrich different truncated or modified Aβ peptides. Mass spectrometric analysis shows unique Aβ peptides that 1) have not been previously reported in other mass spectrometric analyses of AD brains (underlined) 2) uniquely present in AD (bold font) are differentially highlighted. Predicted molecular weights and observed molecular weights have been calculated using ExPASy PeptideMass and ProteinChip Data Manager software (Bio-Rad), respectively. The number of subjects (AD, Alzheimer's disease; PA, Pathological aging; NDC, controls) displaying each uniquely identifiable Aβ peptide and the percentage of occurrence of such peptides corresponding to the total sample size is denoted. Aβ, amyloid β; MW, molecular weight; n, number; SDS, sodium dodecyl sulfate.

**Table 4:** Mass spectrometric analysis of formic acid (FA) fraction immunoprecipitated with Ab9 from human brains reveal unique Aβ peaks.

Aβ Peptide	predicted MW	observed MW	AD (*n *= 16)	PA (*n *= 9)	NDC (*n *= 8)
1-18	2167.28	2167.70	**2 (12.5%)**		
1-26	3020.16	3021.02			***2 (25%)***
pE11-40	3115.65	3151.47			***1 (12.5%)***
1-27	3134.27	3134.77			***1 (12.5%)***
pE11-42	3317.90	3318.91	1 (6.3%)	2 (22.2%)	
9-40	3371.90	3372.97	8 (50%)	6 (66.7%)	5 (62.5%)
1-30 +16 Da	3406.57	3409.22	**1 (6.3%)**		
1-36	4017.42	4019.60	**1 (6.3%)**		
1-37	4074.47	4076.58	**2 (12.5%)**		
1-37 +16 Da	4090.47	4094.07	**1 (6.3%)**		
1-38	4131.52	4134.52	**4 (25.0%)**		
1-38 + 16 Da	4147.52	4150.11	**1 (6.3%)**		
4-42	4198.74	4200.32		*2 (22.2%)*	
4-42 +16 Da	4214.74	4216.78	1 (6.3%)	1 (11.1%)	1 (12.5%)
1-39	4230.65	4232.14	2 (12.5%)	1 (11.1%)	
3-41	4256.85	4260.60	2 (12.5%)	1 (11.1%)	
pE3-42	4309.90	4312.76		*1 (11.1%)*	
1-40	4329.78	4332.14	12 (75.0%)	6 (66.7%)	2 (25%)
1-40 +16 Da	4345.78	4347.41	7 (43.8%)	1 (11.1%)	1 (12.5%)
2-42	4395.93	4399.63			***1 (12.5%)***
1-41	4442.94	4444.51	1 (6.3%)	1 (11.1%)	
1-42	4514.02	4516.58	16 (100%)	9 (100%)	4 (50%)
1-42 +16 Da	4530.02	4532.13	9 (56.3%)	4 (44.4%)	1 (12.5%)
1-43	4615.20	4617.58	4 (25.0%)	3 (33.3%)	1 (12.5%)

Human brain tissue, extracted with 70% FA, was sequentially immunoprecipitated with Ab9 and 4G8 antibodies to enrich different truncated or modified Aβ species. Mass spectrometric analysis shows unique Aβ peptides that 1) have not been previously reported in other mass spectrometric analyses of AD brains (underlined) 2) uniquely present in AD (bold font), PA (italicized font) or controls (bold and italicized font) are differentially highlighted. Predicted molecular weights and observed molecular weights have been calculated using ExPASy PeptideMass and ProteinChip Data Manager software (Bio-Rad), respectively. The number of subjects (AD, Alzheimer's disease; PA, Pathological aging; NDC, controls) displaying each uniquely identifiable Aβ peptide and the percentage of occurrence of such peptide corresponding to the total sample size is denoted. Aβ, amyloid β; MW, molecular weight; n, number.

**Table 5:** Mass spectrometric analysis of formic acid (FA) fraction immunoprecipitated with 4G8 from human brains reveal unique Aβ peaks.

Aβ Peptide	predicted MW	observed MW	AD (*n *= 16)	PA (*n *= 9)	NDC (*n *= 8)
1-18	2167.28	2165.21	**1 (6.3%)**		
1-27	3134.27	3135.30	**1 (6.3%)**		
E11-42	3317.90	3320.02	8 (50.0%)	7 (77.8%)	1 (12.5%)
1-29	3319.49	3320.87	**1 (6.3%)**		
11-42	3335.90	3328.79		*1 (11.1%)*	
9-40	3371.90	3373.39	3 (18.8%)	5 (55.6%)	4 (50%)
8-40	3458.98	3460.65	1 (6.3%)	1 (11.1%)	
1-31	3503.72	3503.55	**1 (6.3%)**		
9-42	3556.08	3557.85	**1 (6.3%)**		
8-42	3643.15	3644.86	5 (31.3%)		1 (12.5%)
7-42	3758.24	3758.89	**2 (12.5%)**		
1-34	3787.09	3786.56	**1 (6.3%)**		
1-35	3918.29	3918.96	**1 (6.3%)**		
4-40	4014.57	4016.85	1 (6.3%)	1 (11.1%)	
1-36	4017.42	4018.25	1 (6.3%)	1 (11.1%)	
5-42	4051.57	4053.59	4 (25.0%)		1 (12.5%)
1-37	4074.47	4075.25	**1 (6.3%)**		
1-38	4131.52	4131.33	1 (6.3%)	1 (11.1%)	
4-42	4198.74	4201.29		5 (55.6%)	2 (25%)
4-42 +16 Da	4214.74	4217.09	2 (12.5%)	1 (11.1%)	
1-39	4230.65	4231.46	**1 (6.3%)**		
E3-42	4309.90	4312.76	4 (25.0%)	3 (33.3%)	1 (12.5%)
1-40	4329.78	4331.85	8 (50.0%)	5 (55.6%)	2 (25%)
1-40 +16 Da	4345.78	4348.09	**4 (25.0%)**		
1-42	4514.02	4516.70	10 (62.5%)	7 (77.8%)	4 (50%)
1-42 +16 Da	4530.02	4533.03			***1 (12.5%)***

Human brain tissue, extracted with 70% FA, was sequentially immunoprecipitated with Ab9 and 4G8 antibodies to enrich different truncated or modified Aβ species. Mass spectrometric analysis shows unique Aβ peptides that 1) have not been previously reported in other mass spectrometric analyses of AD brains (underlined) 2) uniquely present in AD (bold font), PA (italicized font) or controls (bold and italicized font) are differentially highlighted. Predicted molecular weights and observed molecular weights have been calculated using ExPASy PeptideMass and ProteinChip Data Manager software (Bio-Rad), respectively. The number of subjects (AD, Alzheimer's disease; PA, Pathological aging; NDC, controls) displaying each uniquely identifiable Aβ peptide and the percentage of occurrence of such peptide corresponding to the total sample size is denoted. Aβ, amyloid β; MW, molecular weight; n, number.

Several modified Aβ peptides, that were consistent with a 16 Da increase in mass, were observed. This mass shift is typically indicative of oxidation. Control studies demonstrated that the methodology used did not induce oxidation of reduced synthetic Aβ. Thus, there was evidence that Aβ1-30, 1-37, 1-38, 1-40 and 1-42 were present in the brain as oxidized species. In most cases the peptides were detected in only a few samples among the cohort sets and there were no notable differences in the rate of detection between AD, PA and controls. However, oxidized Aβ peptides were detected more frequently in AD cases with CAA (ten of ten) compared to cases without CAA (one of six). This association was not apparent in PA where oxidized Aβ peptides were detected in two of four cases with and three of five cases without CAA. Three peaks, consistent with pyroglutamate Aβ, were detected in the 70% FA fraction of AD, PA and controls: AβpE11-40, AβpE11-42 and AβpE3-42. These species were not detected in the 2% SDS lysates suggesting that they are highly insoluble.

### Immunoblotting analysis of Aβ species

**Figure 8:**
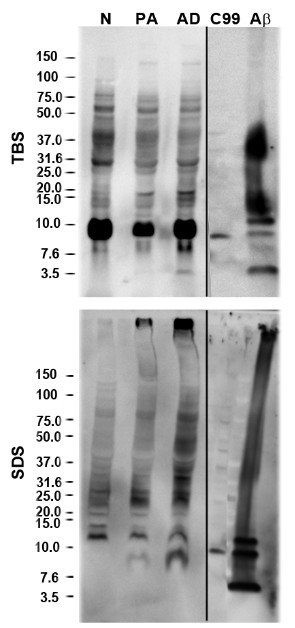
**Immunoblot analysis of Aβ species from sequentially extracted human prefrontal cortical tissue lysates**. Human hippocampi from Alzheimer's disease (AD), pathological aging (PA) and normal (N) cohorts were sequentially extracted with TBS, 2% SDS extracted lysates from human subjects. Representative immunoblots probed with 82E1 antibody are shown. Control lanes includes cell lysates expressing C99 (CTFβ) and recombinant Aβ1-42 (43 pmol). Aβ, amyloid β; SDS, sodium dodecyl sulfate; TBS, Tris buffered saline.

**Figure 9:**
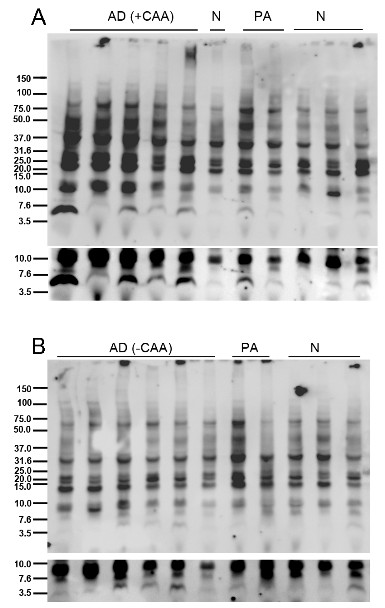
**Immunoblot analysis of Aβ species from RIPA soluble lysates from human subjects**. Human hippocampi from Alzheimer's disease (AD), pathological aging (PA) and normal (N) cohorts were extracted with RIPA. Representative anti-82E1 immunoblot of RIPA extracted lysates of Alzheimer's disease (AD), pathologic aging (PA) and control (N) subjects with (**A**) and without (**B**) a post-mortem clinical pathological diagnosis of cerebral amyloid angiopathy (+CAA). Longer exposure panels below highlight no significant differences between the three cohorts in the dimer/trimer molecular weight range. Aβ, amyloid β; RIPA, radioimmunoprecipitation buffer.

We next analyzed these samples by SDS-PAGE followed by Western Blot. The representative blots shown were separated by electrophoresis, transferred to nitrocellulose, not boiled and stained with 82E1 antibody (anti-Aβ1-16) (IBL). We found that this method gave the most sensitive detection and, based on estimates, our detection limit was 1 to 10 pmol monomeric Aβ. A number of Western Blots failed to differentiate oligomer assemblies between PA and AD in TBS and 2% SDS lysates (Figure 8) and RIPA lysates (Figure 9). We detected a 10 kDa band in the TBS and RIPA lysates of AD, PA and NDC cohorts. This band could be an oligomeric assembly of Aβ, an APP β-C-terminal fragment (since there is cross-reactivity of these two species with 82E1) or a non-specific band. This band was consistently detected with other anti-Aβ antibodies, although these other antibodies had lower limits of sensitivity.

## Discussion

In this study we systematically characterized Aβ levels and solubility, peptide profiles and oligomeric assemblies in a postmortem series of PA, AD and NDC cohorts. Using a panel of Aβ sandwich ELISAs we appraised the levels of Aβ1-40, Aβ1-42 and Aβtotal, as well as Aβx-42. We found extensive overlap in Aβ levels between AD and PA brains. On average, AD and PA lysates contained much more Aβ than NDC samples. Aβ levels in PA lysates were similar to the levels in the AD lysates, ranging from almost equivalent to approximately 50% less than the Aβ detected in AD lysates and solubility profiles were similar with the vast majority of Aβ in PA and AD requiring either SDS or FA to solubilize.

We examined Aβ peptide profiles by IP/MS in AD, PA and NDC patient brain lysates. Aβ1-42 was identified as the predominant peak in the SDS and FA 'insoluble' fractions across all groups. As noted above, it was the only Aβ peptide detected by IP/MS in all PA and AD cases. In addition, several unique NH_2_- and COOH-terminal truncated Aβ peptides were observed in the AD brain lysates. However, few unique truncated Aβ peptides were observed in any one patient group and these peptides represented minor peaks. These truncated peptides may represent alternate cleavage products by β- and γ-secretases or products of Aβ degradation.

Interestingly, we detected peaks that correspond to Aβ1-30, 1-37, 1-38, 1-40 and 1-42 with a 16 Da mass shift that we hypothesize to be oxidation products of Aβ. Oxidation of these Aβ peptides could be an artifact due to the extraction and IP/MS techniques utilized in this study; however, this is unlikely since we did not observe the mass shift in other detected Aβ peptides or in control IP/MS studies using reduced synthetic Aβ. Although Aβ is composed of several amino acids that could be oxidized, most studies have suggested oxidation occurs primarily at the methionine residue at position 35 (Met-35) [40]. A number of studies suggest that oxidized Aβ peptides are present in the brain and that oxidation of Aβ1-42 decreases the rate of aggregation, disrupts fibril morphology and inhibits oligomerization [41,42]. Oxidized Aβ peptides were observed as minor peaks in the spectra, indicating that these represent a minor fraction of total Aβ since control studies showed that both peptides ionized at the same levels. There were no striking differences in oxidized peptides between AD and PA patients. However, remarkably all of the AD patients with CAA had oxidized Aβ peptides while only one of six AD patients without CAA had oxidized Aβ peptides.

We detected peaks corresponding to pyroglutamate modified Aβ (AβpE), at position 11, AβpE11-40, AβpE11-42, and at position 3, AβpE3-42, in the insoluble lysates of AD, PA and NDC. AβpE is formed by glutamate cyclization at position 11 by glutaminyl cyclase [43]. The conversion of glutamate to pyroglutamate is reported to protect the Aβ peptide from degradation through resistance to aminopeptidases [44]. AβpE is also reported to be highly prone to oligomerization and can possibly seed the oligomerization and fibrillation process of full-length Aβ species [18,20,45]. Based on these data as well as other studies examining AβpE levels and animal modeling studies that manipulate AβpE levels, an initiating role for AβpE in AD has been proposed [46-50]. Although our detection of AβpE in a control brain would not be inconsistent with a postulated role in seeding aggregation, its presence in some PA brains suggest that its toxicity is not inherently different from other aggregated Aβ peptides.

There is currently much debate regarding which types of Aβ aggregates are the most toxic. Aβ rapidly forms stable fibrillar amyloid structures, which account for much of the Aβ that accumulates in the AD brain. Over the last decade, a variety of soluble oligomeric species, including dimers, trimers, tetramers and 10-12-mers have been identified and isolated [35,51-53]. These oligomers have been shown to be biologically active as they inhibit hippocampal long-term potentiation and create memory impairments when injected into rodents [35,53,54]. Additional circumstantial data suggests that oligomeric assemblies may account for some of the behavioral deficits observed in APP mice [55]. As there is no standard methodology to detect oligomeric assemblies, we empirically settled on a method that was the most sensitive in our hands, and also would enable us to survey oligomeric assemblies in multiple brain lysates. This survey revealed that there were no obvious differences in higher molecular weight bands between AD, PA and NDC. Our data are consistent with a recent publication that reported a more extensive analysis of oligomeric assemblies and also failed to detect major differences in PA and AD [34]. Indeed, it is not the relatively small increases in the TBS and RIPA extractable Aβ pools but that of the SDS and 70% FA extractable insoluble Aβ pools that clearly distinguish both AD and PA from controls.

## Conclusions

In summary, we investigated Aβ levels, peptides and assemblies from soluble, detergent-soluble and insoluble pools from AD, PA and NDC brain. We found only subtle quantitative differences between PA and AD brains that, in most cases, did not reach significance. We found overlap between the PA and AD Aβ peptide profile, as examined by IP/MS, but AD patients contained additional amino terminal truncated Aβ peptides. There were no major differences observed in SDS-stable Aβ oligomeric assemblies. We cannot rule out the possibility that there are conformational differences or very subtle differences in minor Aβ peptides or assemblies that distinguish AD from PA; however, our data, which shows extensive similarities between deposited Aβ in AD and PA, would indicate that PA is not likely to represent a form of benign Aβ deposition. Indeed, our data are more consistent with the hypothesis that PA represents an initial prodromal stage of AD and that these individuals would eventually go on to develop clinical symptoms, if they live long enough [31,32]. Notably, PA brains do not completely lack cortical tau pathology; however, pathological phospho-tau levels are present in lower levels compared to AD (Figure 1). Indeed, given predictions of the amyloid cascade hypothesis, many of which are being demonstrated in living humans via imaging and cerebrospinal fluid studies, one would predict that a subset of cognitively normal subjects would die with heavy amyloid loads but limited tau pathology [56,57]. This possibility needs to be taken into account in the debate regarding identification of the toxic Aβ species. We should not discount the role of insoluble fibrillar assemblies in driving downstream pathologies; assemblies may produce an insidious form of cellular toxicity that may be difficult to assess in more acute experimental models. This concept may best be phrased that amyloid-like assemblies are necessary but not sufficient to drive neurodegeneration. Although speculative, it is also possible that PA patients may be protected from the downstream effects of Aβ. In the latter case, genetic studies, gene expression profiling, or perhaps even development of induced pluripotent stem cells from PA subjects could identify factors that confer protection from Aβ. Ultimately, in order to determine whether PA represents a prodromal phase of AD or could reflect inherent resistance to Aβ, long-term longitudinal amyloid imaging, biomarker studies, and postmortem neuropathological examination will be needed. If powered sufficiently such studies could determine whether there are subsets of individuals who develop AD-like plaque pathology but retain normal cognitive function without neurodegeneration after extended periods of time or whether parenchymal Aβ accumulation invariably leads to neurodegeneration and AD.

## Abbreviations

Aβ: amyloid-beta; AD: Alzheimer's disease; APP: amyloid β protein precursor; CAA: cerebral amyloid angiopathy; ELISA: enzyme-linked immunosorbent assay; FA: formic acid; HRP: horseradish peroxidase; IP/MS: immunoprecipitation/mass spectrometry; mAb: monoclonal antibody; PA: pathological aging; NDC: non-demented controls; RIPA: radioimmunoprecipitation buffer; SDS: sodium dodecyl sulfate; TBS: Tris buffered saline.

## Competing interests

The authors declare that they have no competing interests.

## Authors' contributions

BDM, TEG and PD conceived and designed the study. DWD provided human samples and contributed to critical discussions. BDM, PC, AMB, TM and TL prepared samples and acquired data. BDM and TEG drafted the manuscript. BDM, PC, YL, TK, PD and TEG contributed to the interpretation of findings. All authors read and approved the final manuscript.

## Supplementary Material

Additional file 1 **Figure S1**. Biochemical analysis of Aβ levels from human brain lysates. A panel of sandwich ELISAs measuring Aβ1-40, Aβ1-42, Aβtotal and Aβx-42 from brain lysates sequentially extracted with TBS (A), RIPA (B), 2% SDS (C) and 70% formic acid (D) is shown. Data are presented as scatter dot plots, *n *= 16 (AD), 8 (PA) and 6 (NDC). (***P < 0.001, **P < 0.01, *P < 0.05 by ANOVA with tukey post-hoc analysis raw data analyzed (A, B) and log-transformed data analyzed (C, D)). Click here for file
