# Supplementary figures and images for: Overlapping profiles of Aβ peptides in the Alzheimer's disease and pathological aging brains
Source: Alzheimers Res Ther. 2012 May 23;4(3):18. doi: 10.1186/alzrt121 (PMC3506932; doi:10.1186/alzrt121)

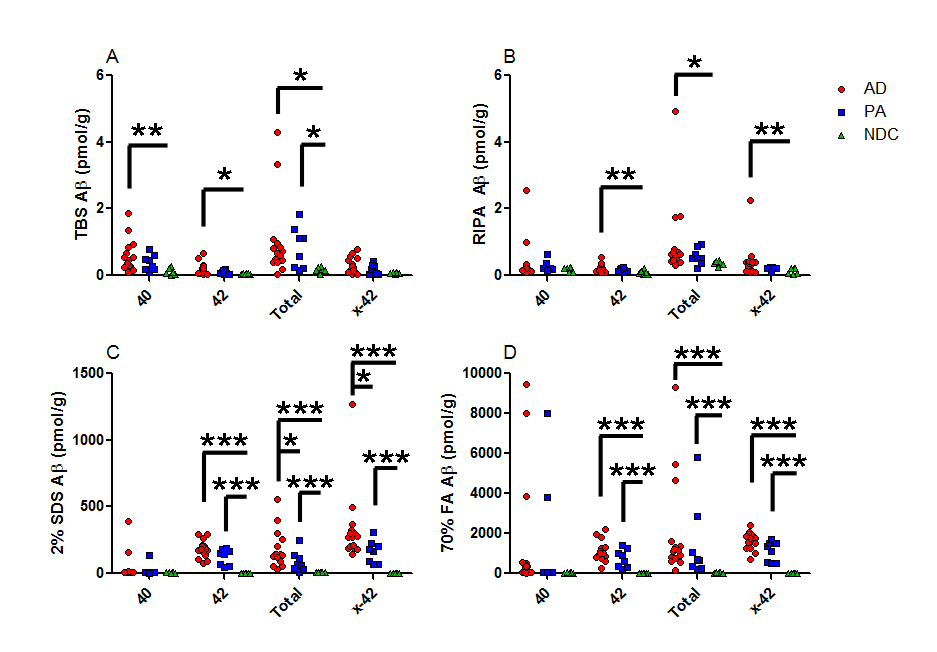

Supplement: Additional file 1 — Figure S1. Biochemical analysis of Aβ levels from human brain lysates. A panel of sandwich ELISAs measuring Aβ1-40, Aβ1-42, Aβtotal and Aβx-42 from brain lysates sequentially extracted with TBS (A), RIPA (B), 2% SDS (C) and 70% formic acid (D) is shown. Data are presented as scatter dot plots, n = 16 (AD), 8 (PA) and 6 (NDC). (***P < 0.001, **P < 0.01, *P < 0.05 by ANOVA with tukey post-hoc analysis raw data analyzed (A, B) and log-transformed data analyzed (C, D)). [file alzrt121-S1.TIFF]
